# Lysogenization of a Lactococcal Host with Three Distinct Temperate Phages Provides Homologous and Heterologous Phage Resistance

**DOI:** 10.3390/microorganisms8111685

**Published:** 2020-10-29

**Authors:** Sofia Ruiz-Cruz, Elvina Parlindungan, Andrea Erazo Garzon, Mona Alqarni, Gabriele A. Lugli, Marco Ventura, Douwe van Sinderen, Jennifer Mahony

**Affiliations:** 1 School of Microbiology & APC Microbiome Ireland, University College Cork, Western Road, Cork T12 YT20, Ireland; sofia.ruizcruz@ucc.ie (S.R.-C.); elvina.parlindungan@ucc.ie (E.P.); 114224203@umail.ucc.ie (A.E.G.); 111220242@umail.ucc.ie (M.A.); 2 Laboratory of Probiogenomics, Department of Chemistry, Life Sciences and Environmental Sustainability, University of Parma, 43121 Parma, Italy; gabrieleandrea.lugli@unipr.it (G.A.L.); marco.ventura@unipr.it (M.V.)

**Keywords:** bacteriophage, *Lactococcus lactis*, prophage, phage-resistance, abortive infection

## Abstract

*Lactococcus lactis* is the most widely exploited microorganism in global dairy fermentations. Lactococcal strains are described as typically harboring a number of prophages in their chromosomes. The presence of such prophages may provide both advantages and disadvantages to the carrying host. Here, we describe the deliberate generation of three distinct lysogens of the model lactococcal strain 3107 and the impact of additional prophage carriage on phage-resistance and anti-microbial susceptibility. Lysogen-specific responses were observed, highlighting the unique relationship and impact of each lysogenic phage on its host. Both homologous and heterologous phage-resistance profiles were observed, highlighting the presence of possible prophage-encoded phage-resistance factors. Superinfection exclusion was among the most notable causes of heterologous phage-resistance profiles with resistance observed against members of the *Skunavirus*, P335, P087, and 949 lactococcal phage groups. Through these analyses, it is now possible to identify phages that may pursue similar DNA injection pathways. The generated lysogenic strains exhibited increased sensitivity to the antimicrobial compounds, nisin and lysozyme, relative to the parent strain, although it is noteworthy that the degree of sensitivity was specific to the individual (pro)phages. Overall, the findings highlight the unique impact of each prophage on a given strain and the requirement for strain-level analysis when considering the implications of lysogeny.

## 1. Introduction

Industrial dairy fermentations rely heavily on the repeated and reliable application of desirable starter cultures. Strains of *Lactococcus lactis* are among the most intensely applied starter cultures in mesophilic fermentations. Most genomes of lactococcal strains are reported to harbor one or more prophages [1,2,3,4]. Because phages pose a threat to consistent fermentation regimes, they have traditionally been viewed as a problematic entity. However, (partial) culture lysis releases intracellular enzymes involved in flavor and aroma development and accelerated cheese ripening [5]. This culture lysis may be mediated by autolysis via AcmA (and its homologues), the major lactococcal glucosaminidase that hydrolyses N-acetylmuramyl-1,4-β-N-acetylglucosamine bonds in peptidoglycan [6,7], or via induction of resident prophages by ultraviolet light or thermal shock. Furthermore, environmental stressors including pH or nutritional starvation may also induce prophage excision and release.

Prophages of *Lactococcus lactis* belong to the heterogenous group of phages termed the P335 quasi-species. The P335 group of phages includes both virulent and temperate members that have recently been classified into five sub-groups based on their genetic composition and morphology [1,8]. Detailed characterization of the model lactococcal strain IL1403 and its prophage-cured derivatives demonstrated the higher susceptibility of the prophage-carrying derivatives to cell wall-targeting and anti-microbial compounds, such as nisin and lysozyme, compared to the prophage-cured version(s). However, the stationary phase survival at 30 °C and thermal stability (at 37 °C) was observed to be higher in the parent strain relative to the prophage-cured derivatives [9]. Therefore, prophage carriage in IL1403 has both positive and negative connotations for the host with important implications for the industrial application of prophage-carrying lactococcal strains. The risk of prophage induction was assessed among a panel of lactococcal strains using mitomycin C as an inducing agent and with subsequent validation by flow cytometry [1]. One in six strains among the 24 strains tested were shown to have inducible prophaes under the conditions assessed, highlighting the relatively stable integration of these prophages in lactococcal strains. Additionally, this finding highlights the high incidence of prophage carriage among lactococcal strains, many of which may be defective or non-inducible (although additional analysis would be required to confirm this), suggesting that they confer a benefit to the host and are thus retained.

Integration of a prophage typically culminates in immunity against secondary infecting homologous phages (by a phenomenon called superinfection immunity, Sii). Constitutive expression of repressor proteins confers a Sii phenotype against phages harboring a homologous repressor protein [10,11]. For lactococcal phages this concept was verified using a repressor from the lytic P335 phage 31, which was shown to confer Sii against a number of related, yet genetically distinct P335 [12].

Certain temperate lactococcal phages encode phage-resistance systems including superinfection exclusion (Sie) systems that prevent DNA injection of heterologous phages [13,14]; abortive infection (Abi) systems that prevent phage development; and methyltransferases, which may protect the resident prophages through modification of the recognition sequence of certain restriction enzymes [4]. The first described lactococcal superinfection exclusion (Sie) system was that of the temperate P335 phage, Tuc2009 (Sie_2009_). This system was shown to provide resistance against certain skunaviruses (formerly called the 936 group) and with limited impact on ceduoviruses (formerly called the c2-like phages) and P335 phages [14]. Sie_2009_ is a small membrane-associated protein with a hydrophobic amino terminus. These characteristics were used to identify additional and distinct lactococcal Sie proteins encoded by genes in the lysogeny modules of prophages of the model strains IL1403 and MG1363 [13]. In addition to these Sie systems, two component systems incorporating a membrane-associated protein and a protein containing a zinc-binding motif similar to metalloproteases acting synergistically have also been identified [14]. One such system named Sie_F7/2AB_ conferred a moderate phage resistance phenotype and significant plaque size reduction against the tested skunaviruses [14]. 

Genome analysis of the predicted prophages of 30 lactococcal strains identified nine strains (and ten prophages) that encode likely Abi systems based on AT% content of individual genes and sequence relatedness to previously defined Abi systems [1]. However, the functionality of these systems remains to be established in cases where sequence identity is not high. The presence of such phage-resistance systems may present a significant benefit to the host through protection against secondary infecting phages. *Lactococcus lactis* subsp. *cremoris* 3107 is a lactococcal strain that is sensitive to phages belonging to a number of lactococcal phage groups, including members of the *Skunavirus*, P335, 949 and P087 phage groups, rendering it a prototypical strain to evaluate impacts on phage sensitivity and models of phage infection [15,16,17]. *L. lactis* strain 3107 is host to a number of temperate P335 phages making it a particularly useful model strain to evaluate the effect of lysogeny. While the interactions of this strain with virulent phages is well interrogated and described, the impact of lysogeny on this strain has not been evaluated. Therefore, the present study aimed to evaluate the genome of *L. lactis* 3107 for the presence and stability of innate prophages, and also assess the impact of integration of three genetically distinct temperate P335 phages on the host in terms of phage-resistance, sensitivity to anti-microbials, and mitomycin C treatment.

## 2. Materials and Methods

### 2.1. Bacteria and Bacteriophage Preparation

Lactococcal strains used in this study are listed in Table S1 and were grown without agitation at 30 °C in M17 broth (Oxoid Ltd., Hampshire, UK.) supplemented with 0.5% glucose (GM17). Lactococcal phages were propagated in GM17 broth cultured with the host strain (3107) at an approximate OD_600 nm_ of 0.2 and supplemented with 10 mM CaCl_2_. SM buffer (10 mM CaCl_2_, 100 mM NaCl, 10 mM MgSO_4_, 50 mM Tris-HCl at pH 7.5) was used as the diluent in all phage assays.

### 2.2. Phage Enumeration Assays

To determine the phage sensitivity profile of *L. lactis* 3107 and its derivatives, solid and semi-solid agar was prepared using GM17 broth supplemented with 1.5% (*w*/*v*) or 0.7% (*w*/*v*) bacteriological agar, respectively. A quantity of 10 mM CaCl_2_ and 0.5% glycine (*w*/*v*) was also incorporated where necessary, and the solid and semi-solid agar plates were employed according to a previously described method [18]. To perform spot assays, an overlay was created by seeding semi-solid agar with the relevant indicator/test strain and spotting 10 µL of 10^8^ pfu/mL phage lysate on the overlay. The plates were incubated overnight at 30 °C. Plaque assays were performed using the previously described double agar approach [18]. The plates were incubated overnight at 30 °C. The efficiency of plaquing (E.O.P.) was determined as the ratio of the titer obtained from the test strain to that of the control strain (*L. lactis* 3107).

### 2.3. Induction Trials of L. lactis 3107

To determine if the prophages of 3107 could be induced using mitomycin C as an induction agent, 50 mL GM17 broth was inoculated with 1% of a fresh overnight culture. This was incubated at 30 °C until an OD_600 nm_ of 0.2 was reached, at which point mitomycin C was added to a final concentration of 0, 0.2, 2, and 3 µg/mL as described in previous studies [1,3]. The OD_600 nm_ was monitored hourly for 16 h. Culture lysis was indicated by a drop in OD values. A positive control of *L. lactis* NZ9000 harboring TP901-1erm was included to verify the lysis phenotype. To ascertain if prophage induction had genuinely occurred, the supernatants/treated cultures of *L. lactis* 3107 and NZ9000 were cleared by centrifugation at 5000× *g* and filtered through a 0.45 µm filter and treated with DNAse at 37 °C for 30 min to degrade host chromosomal DNA. The (potential) phage DNA was extracted using the Norgen Biotek Phage DNA isolation kit and the resulting DNA extract (where present) restricted EcoRV as previously described [1].

### 2.4. Generation of Lysogens of TP901-1, LC3 and Dub35A in L. lactis 3107

A fresh overnight culture (200 µL) of *L. lactis* 3107 was mixed with 10 µL lysate containing approximately 10^7^ pfu of each of the phages (TP901-1, LC3 or Dub35A) which was selected for lysogen preparation. This mixture was transferred to the molten overlay agar and poured onto solid agar plates. The plates were incubated for up to 48 h until visible colonies formed in the overlay. Ten colonies of each was selected and inoculated in 10 mL fresh GM17 broth, incubated overnight at 30 °C and tested for maintenance of phage resistance against the respective phages that were initially used to generate the lysogens/phage-resistant derivatives. The isolates were passaged at least three additional times to select for those with a stable phage-resistance phenotype. The majority were observed to maintain the lysogens with at least eight of the ten derivatives of each strain maintaining the phage-resistance phenotype. One of each of those that retained the phage-resistant phenotype were checked for the presence of phage-specific amplicons by PCR and a representative lysogen of each of the three phages was selected for further analysis. The selected and confirmed 3107-derived lysogens of TP901-1 (3107-TPlys), Dub35A (3107-Dublys), and LC3 (3107-LC3lys) were evaluated for phage sensitivity against a panel of 3107-infecting phages representing four different phage groups (Table S1) using the double agar plaque assay [18].

### 2.5. Lysozyme and Nsin Susceptibility Assays

The susceptibility of *L. lactis* 3107 and its lysogenized derivatives 3107-TPlys, -Dublys, and -LC3lys to antimicrobials was determined using the method described by Aucouturier et al. (2018). Briefly, overnight cultures of 3107 and its derivative strains were diluted 10-fold in Ringer’s solution (Sigma, St. Louis, MO, USA). Five μL drops of the dilutions were spotted on GM17 agar containing different concentrations of inhibitors: from 0 to 0.5 mg/mL of lysozyme (Sigma) and from 0 to 0.3 mg/mL of nisin (Sigma). Plate counts were performed after 36 h of incubation at 30 °C. All experiments were performed at least in triplicate.

### 2.6. DNA Extraction and Genome Sequencing, Assembly and Annotation

DNA was extracted from a fresh 10 mL overnight culture of *L. lactis* 3107-TPlys, -Dublys, and -LC3lys using the Invitrogen Purelink genomic DNA extraction kit according to the manufacturer’s instructions. Chromosomal DNA extracted from each strain was sequenced by the commercial sequencing service provider Probiogenomics (University of Parma, Italy) using an Illumina MiSeq platform. Genomic libraries were constructed using the TruSeq DNA PCR-Free LT Kit (Illumina^®^) and 2.5 μg of genomic DNA, which was fragmented with a Bioruptor NGS ultrasonicator (Diagenode, USA) followed by size evaluation using Tape Station 2200 (Agilent Technologies, Santa Clara, CA, USA). Library samples were loaded into a Flow Cell V3 600 cycles (Illumina^®^) and draft genome Illumina sequencing was performed on a MiSeq genomic platform (Illumina, Cambridge, UK) at GenProbio srl (Parma, Italy). Fastq files of the paired-end reads obtained from the genome sequencing were used as input for genome assemblies through the MEGAnnotator pipeline in default mode [19]. The MIRA program (version 4.0.2) was used for de novo assembly of genome sequence data [20]. Following final genome assembly, putative protein-encoding genes were identified using the prediction software Prodigal (version 2.0) [21]. Protein-encoding genes were automatically annotated using a BlastP v2.2.26 (cut-off E-value of 0.0001) sequence alignments against the non-redundant protein (nr) database curated by NCBI (ftp://ftp.ncbi.nih.gov/blast/db/). Subsequently, using the multiple genome alignment software Mauve v2.4.0 [22], a comparative genome analysis of *L. lactis* 3107 (i.e., the reference genome) and its derivatives was undertaken to establish the location of the incorporated prophages in the particular lysogen-derived genomes.

### 2.7. Bioinformatic Analyses

To ascertain if the genome of *L. lactis* 3107 harbors (predicted) prophages, the sequence was interrogated using Phaster [23]. Using the output of the analysis, the predicted intact, partial (i.e., incomplete), and questionable phage sequences were inspected manually to determine the prophage-like regions for phage-related functions and confirmation of the automated outputs.

The bacterial/phage attachment sites (*attB/P*) required for integration of TP901-1 and LC3 had previously been established [24,25]. For TP901-1, the *attB* site was confirmed as a 13 bp sequence (5′-tcaatcaaggtaa-3′), whereas for LC3, a core 9 bp sequence (5′-ttcttcatg-3′) was identified in its presumed *attP*. For Dub35A, the attachment sites had not previously been identified, thus the genome sequence was analyzed using Phaster [23]. The genome of *L. lactis* 3107 was investigated using BlastN for the presence of the associated *attB* sites to establish the number of possible insertion sites.

The genomes of the P335 phages that infect *L. lactis* 3107 (listed in Table S1) were downloaded from the NCBI database (https://www.ncbi.nlm.nih.gov/). The GenBank accession numbers of the analyzed phages are as follows: AF304433.1 (TP901-1); AF242738.3 (LC3); NC_031017.1 (63301); KX160220.1 (Dub35A); KX160219.1 (C41431); KX456207.1 (50101); KX456212.1 (86501). The lysogeny modules of the temperate *L. lactis* 3107-infecting phages were compared using BlastN and BlastP analysis. To identify potential phage-resistance systems encoded by Dub35A, each gene was analyzed for AT% content and the gene products were analyzed using HHPred [26], Pfam [27], and BlastP.

## 3. Results

### 3.1. L. lactis 3107 Harbours Stable Resident Prophages

**Figure 1:**
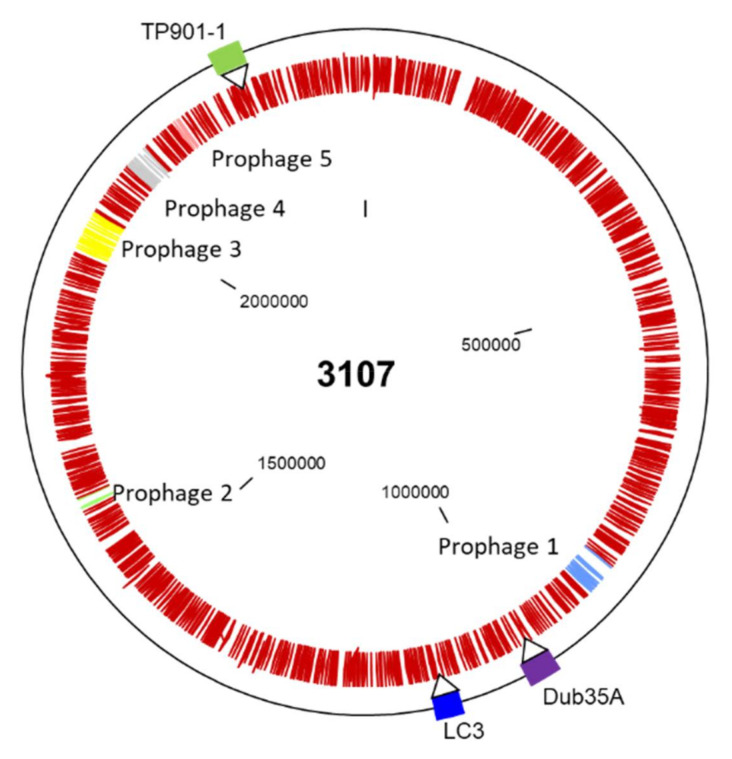
Genome map of *L. lactis* 3107 with the native predicted prophage-encoding regions highlighted in light blue (prophage 1), bright green (prophage 2), yellow (prophage 3), grey (prophage 4), and pink (prophage 5) within the chromosome of the host. The outer ring is punctuated with markers to indicate the position of Dub35A, LC3, and TP901-1 in the genomes of their respective lysogens relative to the parent strain.

To establish the incidence of prophage(s) in the genome of *L. lactis* 3107, the sequence was analyzed using the Phaster server. This analysis predicted the presence of two intact prophages, two incomplete and one questionable prophage region (Table 1, Figure 1). The identified prophages bear some sequence similarity to prophages of several lactococcal strains including those of the prototypical lactococcal strains IL1403 (bIL309, bIL310; Table 1) and MG1363 (TP712; Table 1).

Prophage regions 1 and 3 are predicted to be intact and manual inspection of their genome content supports this with clearly identifiable structural and replication functions in both prophage regions. Furthermore, prophage region 1 is predicted to encode an AbiD1-like protein based on BlastP and BlastN analysis. This is also in agreement with the elevated AT% content of this gene (69.5%) relative to the overall genome AT% content (64.09%), a characteristic of Abi systems [28]. Readily identifiable phage-resistance systems were observed on prophage region 1.

Prophage region 2, regarded by Phaster as a questionable prophage region, appears to comprise many phage-related functions and proteins including a predicted integrase, anti-repressor, holin, and replication/DNA binding proteins. However, it appears to lack genes encoding the structural components of a phage, indicating that this is likely an incomplete phage. However, it should be noted that such satellite phages that do not encode structural components may use so-called “helper phages” to form intact phage particles in other species [29]. Similarly, for prophage region 4, ancestral phage-related components are present, although with limited morphogenesis-related functions. Interestingly, this prophage region is predicted to encode an Abi, albeit seemingly truncated, in addition to a HsdS specificity subunit of a Type I restriction-modification system. Prophage region 5 possesses only one gene with possible phage ancestry and is thus not considered further as a prophage component in this strain.

To establish if these prophages were integrated stably or if they could be readily induced from the host genome, actively growing cultures were challenged with 0, 0.2, 2, and 3 µg/mL mitomycin C and the OD_600 nm_ monitored hourly over a period of 16 h. The growth of the strain was not impacted significantly in the presence of any tested concentration of mitomycin C in this study. To ensure that prophage induction had not occurred in the absence of visible lysis, a DNA extraction of the cell free supernatant of mitomycin-treated *L. lactis* 3107 and the positive control *L. lactis* NZ9000 was undertaken. The resulting extract was treated with EcoRV to generate a restriction profile of the (potentially) induced phage DNA. Whereas the NZ9000 supernatant yielded discernible bands as expected, the DNA extract of the 3107 supernatant did not yield a discernible band before or after EcoRV treatment, suggesting that limited, if any, prophage induction had occurred. Therefore, it was assumed that the resident prophages are stably integrated in the chromosome of the host and were not expected to interfere with downstream prophage integration studies.

### 3.2. Bioinformatic Analysis of 3107-Infecting Temperate Phage Lysogeny Modules

*L. lactis* subsp. *cremoris* 3107 is sensitive to infection by members of several lactococcal phage groups, including the P335, 936, 949, and P087 phages. Among these, only the P335 phage group is reported to include temperate members. In the present study, seven P335 phages capable of infecting *L. lactis* 3107 were analyzed for the presence of lysogeny-related functions and compared to identify different lysogeny module genotypes. This cohort of phages was comprised of TP901-1, LC3, Dub35A, C41431, 50101, 63301, and 86501. Typically, the lysogeny module of temperate (lactococcal) phages encodes an integrase that facilitates recombination between the bacterial (*attB*) and phage (*attP*) attachment sites, and a repressor function for the maintenance of lysogeny. In some cases, additional genes may be present between the integrase- and repressor-encoding genes, some of which have previously been demonstrated to provide protection against heterologous phages (called superinfection exclusion, Sie). Here, we evaluated the presence of additional genes in the lysogeny modules of the temperate phages that may encode Sie functions or indeed (as yet) undefined functions. Through this analysis, four genotypes of lysogeny modules were identified, i.e., the TP901-1, LC3 (LC3, 50101 and 86501), Dub35A, and 63301 types, and each is described in more detail below.

It has previously been reported that 50101, 86501, and 63301 bear the greatest sequence similarity to the P335 subgroup III phage LC3 which is in agreement with phylogenetic analysis of the whole nucleotide sequences of the seven phages applied in this study. Furthermore, Dub35A and C41431 were previously grouped among the P335 subgroup III phages, whereas TP901-1 possesses a distinct morphology with a large adhesion device, termed a baseplate, at the tail tip and is grouped among the P335 subgroup II phages based on its morphology and DNA sequence [1,8].

**Figure 2:**
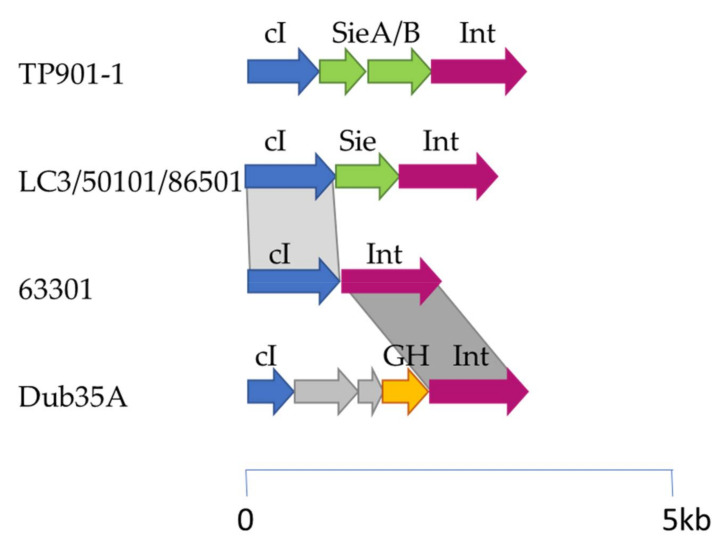
Schematic representation of the lysogeny modules of the 3107-infecting phages TP901-1, LC3, 50101, 86501, 63301, and Dub35A. Blue arrows represent cI repressor-encoding genes, green arrows represent Sie-encoding genes, pink represent integrase functions, orange arrows represent a glycosyl hydrolase, and the grey arrows represent genes encoding proteins of unknown function. Grey shaded regions signify regions of homology between the indicated gene products with light grey indicating 38% aa identity and dark grey indicating 51% aa identity. The scale is indicated under the schematic.

Among the seven P335 phages analyzed, C41431 was previously characterized as a virulent P335 phage because its genome lacks an integrase-encoding gene, although a temperate ancestry is suggested by the presence of putative repressor- and antirepressor-encoding genes. The remaining six phages all appear to possess intact lysogeny modules with at least the integrase- and repressor-encoding genes. LC3, 50101, and 86501 all share identical repressor- (98–100% id), integrase- (100% identity) and Sie- (100% id) encoding genes to each other and those of the temperate lactococcal phage Tuc2009. Phage 63301 genome encodes a repressor with 38% id to those of Tuc2009, 50101, 86501, and LC3, and does not appear to encode an Sie function in its genome (Figure 2). TP901-1 and Dub35A genomes each encode a cI repressor that is unique among 3107-infecting phages. In addition to the cI repressor, the genome TP901-1 also harbors two additional genes (*orf 2, orf3*) in its lysogeny module. Their gene products bear 100% aa identity to those of *L. lactis* F7/2, which have been shown to effect a reduction in plaque size against skunaviruses when expressed in tandem [14]. The lysogeny module of Dub35A harbors three genes between the integrase and repressor-encoding genes. These are predicted to encode a glycoside hydrolase (*orf3*) and proteins of unknown function (*orf4, orf5*). TMHMM transmembrane modelling of the three proteins did not identify any membrane-association, a feature that is common among previously characterized lactococcal superinfection exclusion systems. For the purpose of this study, phages TP901-1, Dub35A, 63301, and LC3 were selected as representatives of the distinct lysogeny module types for the 3107-infecting temperate phages.

### 3.3. Prophage Carriage Endows Homologous and Heterologous Phage Resistance

To evaluate the impact of prophage carriage on phage sensitivity/resistance against a range of 3107-infecting phages, three separate lysogens were generated representing those with distinct lysogeny module components, i.e., a lysogen of TP901-1 (3107-TPlys), LC3 (3107-LC3lys), and Dub35A (3107-Dublys). Despite several attempts to generate lysogens of 63301, none were isolated in this study. It is tempting to speculate that the phage integrase is no longer functional, possibly due to the incorporation of mutations in its genome after its excision from the original host. The three lysogens were tested against a panel of 3107-infecting phages and representing members of the *Skunavirus* (formerly termed the 936 group), P335, 949, and P087 lactococcal phage groups.

**Figure 3:**
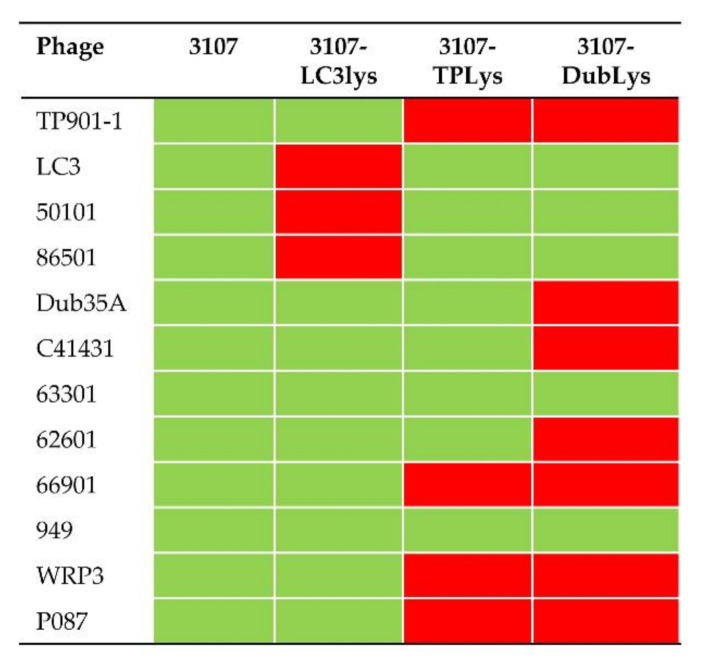
Matrix diagram of the phage resistance (red) or sensitivity (green) of 3107 and its three lysogenized derivatives 3107-LC3lys, 3107-TPlys, and 3107-Dublys. A strain is considered phage sensitive when the E.O.P. value was in the range of 10^−2^–1 and E.O.P. values of less than 10^−2^ were considered phage-resistant.

**Figure 4:**
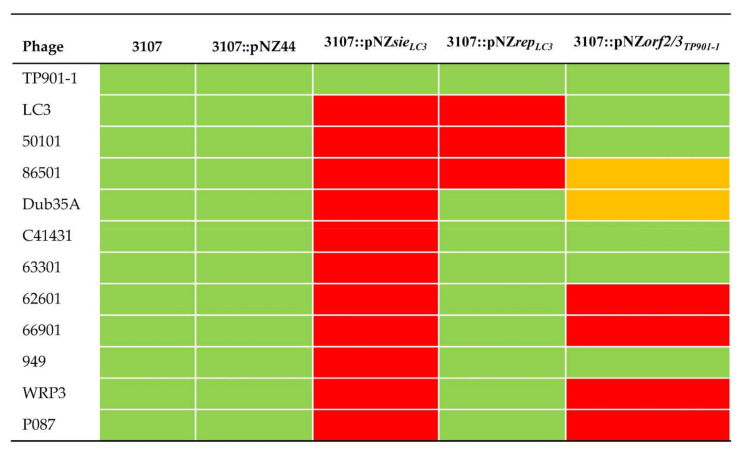
Matrix diagram of the phage resistance (red), partial sensitivity (orange), or sensitivity (green) to the phages tested in this study against *L. lactis* 3107 and its derivatives harboring plasmids expressing *sie_LC3_, rep_LC3_, orf.*

Lysogen 3107-TPlys was observed to exclude secondary infecting TP901-1 while most other phages were largely unaffected by the presence of this prophage (Figure 3, Table S2). A notable observation was the reduction in plaque size and phage numbers for the skunaviruses 62601 and 66901. This observation is consistent with previous studies of superinfection exclusion systems encoded by lactococcal prophages [14]. In a previous report, a dual component system identified in a prophage of *L. lactis* F7/2, termed Sie_F7/2AB_, caused a reduction in plaque size against skunaviruses. Sie_F7/2AB_ proteins are 100% identical to Orf2/3 of TP901-1 and were presumed to exert a similar effect. To assess this notion, the *orf2/3*_TP901-1_ genes were cloned into pNZ44 and the resulting recombinant plasmid, designated pNZ*orf2/3_TP901-1_*, was then transformed into *L. lactis* 3107 to generate strain 3107:: pNZ*orf2/3_TP901-1_*, which was then tested for its sensitivity against various phages. Both the E.O.P. and plaque size were reduced for phages 62601 and 66901, and complete resistance was observed against P087 and WRP3. These data confirm that the observed resistance and plaque morphology changes were due to expression of *orf2/3*_TP901-1_ (Figure 4, Table S3).

Lysogen 3107-LC3lys was shown to exhibit a phage-resistance phenotype against those phages possessing homologous cI repressors (50101 and 86501) (Figure 3, Table S2). To ascertain the molecular components that underpin the observed resistance, the repressor- and Sie-encoding genes of LC3 (*rep_LC3_* and *sie_LC3_*) were cloned and transformed into *L. lactis* 3107 and tested for phage sensitivity (Table S3, Figure 4). Notably, phages LC3, 50101, and 86501 encode homologous equivalents of *sie_2009_* (Sie-encoding gene of the P335 phage Tuc2009) with 100% identity. This system has previously been shown to provide protection against certain skunaviruses.

As expected, expression of *rep_LC3_* effected phage-resistance against phages of the P335 group that shared a homologous repressor (86501, 50101, LC3; Figure 4, Table S3), without affecting sensitivity to the skunaviruses or members of the 949 or P087 groups. In contrast, overexpression of *sie_LC3_* in *L. lactis* 3107 effected complete resistance against all phages tested in this study, with the exception of TP901-1, for which a 2-log reduction in E.O.P. was observed (Table S3). This highlights the broad effect of this Sie system against lactococcal phages and which is considerably broader than was previously known.

Lysogen 3107-Dublys exhibited phage resistance against multiple phages belonging to the P335 group (TP901-1, Dub35A), *Skunavirus* (62601, 66901), and the 949 group (WRP3) (Table S2). Because the putative cI repressor of Dub35A is unique among the phages applied in this study, the broad range of resistance afforded by the integration of Dub35A in the host chromosome cannot be explained by the activity of phage exclusion via the repressor. Indeed, obvious phage-resistance genes such as superinfection exclusion systems were not identified in the lysogeny module. Therefore, the three genes encoded between the integrase and repressor genes were cloned individually or in combination (in the case of *orfs4/5*) to ascertain if expression of these genes alone or in combination underpins the observed phage-resistance. However, expression of *orf3_Dub35A_, orf4_Dub35A_, orf5_Dub35A_*, or the combination of *orf4/5_Dub35A_* did not affect the sensitivity of the host strain *L. lactis* 3107 to any of the tested phages. Consequently, the genome of Dub35A was scrutinized in detail to identify genes beyond the lysogeny module that may impart such phage-resistance. Based on BlastN and BlastP analysis, no obvious candidates could be identified. However, based on AT% content analysis of each gene, one candidate (*orf12_Dub35A_*) was identified that exhibited 65 AT% content and that displays sequence homology to Gp157 of *Streptococcus thermophilus* Sfi phages, which was previously suggested to confer phage-resistance by interfering with phage replication [30]. This protein is conserved in lactococcal and dairy streptococcal phage replication modules, particularly those of temperate phages [31]. Therefore, *orf12_Dub35A_* was cloned in pNZ44 and transformed into *L. lactis* 3107 and challenged against the panel of phages. However, only very modest effects were observed on the strain in terms of phage-resistance, indicating that this gene is either not responsible for the observed phenotype or is required in combination with additional factors that are encoded on the genome of Dub35A. To ascertain if the observed resistance was due to chromosomal mutations or insertion/deletion (indels) events, the genome sequence of the lysogen was analyzed for the presence of single nucleotide polymorphisms (SNPs) and indels; however, no such SNPs or indels were identified.

### 3.4. Location of Lysogens in Host Genome

The attachment site of TP901-1 in *L. lactis* 3107 has previously been defined as a 13 bp sequence (TCAATCAAGGTAA) with a single incidence in the host genome [24]. The core sequence of the attachment site for LC3 was confirmed as a 9 bp sequence (TTCTTCATG) [25] present in 29 identical copies on the *L. lactis* 3107 genome based on BlastN analysis. Phaster analysis of the genome of Dub35A identified a 12 bp *attP* site (CATACGAAATTAC). Two iterations of 11 of the 12 bases of this sequence occur once in the genome of *L. lactis* 3107 (either omitting the first or the last base) based on BlastN analysis. To confirm that the lysogens were intact insertions of the complete genomes of the temperate phages and to evaluate and confirm the positions and directionality of the inserted prophages, the genomes of the generated lysogens were sequenced. All lysogens harbored intact prophages, with TP901-1 inserting its genome within *l3107_2234*, which is predicted to encode the competence-associated function ComGC, truncating its protein sequence in a leftward orientation (Figure 1). This location is in agreement with the location of the *attB* site identified at position 2,204,582–94 on the chromosome of *L. lactis* 3107. The genome of LC3 was observed to be inserted between *l3107_1089* and *l3107_1090*, which are predicted to encode a DNA repair protein and a redox-sensing transcriptional repressor, respectively (Figure 1). LC3 is integrated in a rightward orientation. Dub35A is integrated between *l3107_0971* and *l3107_0972,* which are predicted to encode DNA gyrase subunit B and a protein of unknown function, respectively, and with a rightward orientation (Figure 1).

### 3.5. Lysogen-Specific Impacts of Antimicrobial Compounds

**Figure 5:**
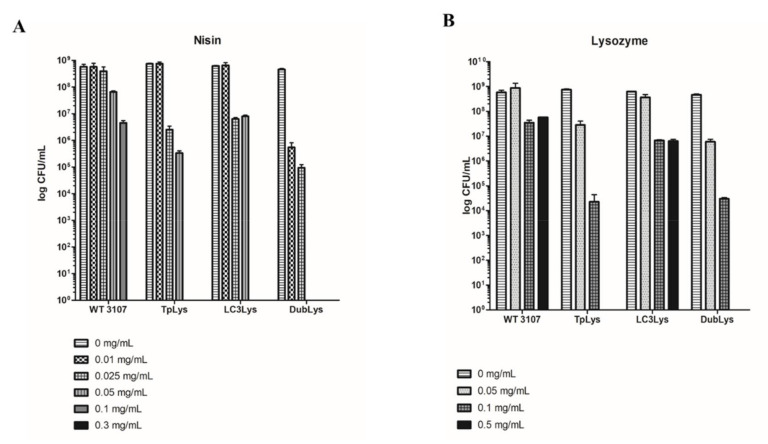
Graphical representation of the susceptibility of *L. lactis* 3107 and its derivatives 3107-TPlys, -LC3lys, and -Dublys to nisin (panel **A**) at concentrations of 0–0.3 mg/mL, and lysozyme (panel **B**) at concentrations of 0–0.5 mg/mL.

In previous studies, antimicrobial susceptibility was observed in general to be higher in the presence of resident prophages than in prophage-cured derivatives [9]. In the present study, a complementary approach was taken whereby lysogens were generated and evaluated for antimicrobial susceptibility where newly acquired prophages have been integrated in the host chromosome. Each of the three lysogens generated in the present study, in addition to the parent strain, 3107, were tested against nisin and lysozyme at a range of concentrations. The lysogen 3107-Dublys appeared most sensitive to both antimicrobials with a 2-log reduction in viable cell numbers in the presence of just 0.05 mg/mL lysozyme compared to the parent strain and a 3-log reduction in cell numbers with 0.01 mg/mL nisin (Figure 5). Furthermore, complete inactivation of the lysogen was observed at 0.5 mg/mL lysozyme and 0.05 mg/mL nisin. The lysogen 3107-TPlys exhibited similar sensitivity as 3107-Dublys to lysozyme but greater resistance to nisin (2-log greater resistance). The lysogen 3107-LC3lys appeared to be the most resistant of the three lysogens to the tested antimicrobials, although it displayed higher sensitivity to both antimicrobials than the parent strain (Figure 5). These findings highlight lysogen-specific responses to external pressures exerted by antimicrobial compounds.

## 4. Discussion

Prophage carriage among lactococci is a widespread phenomenon and may impart both positive and negative attributes in an industrial context. In the present study, it was aimed to evaluate the impact of this relationship through the generation of lysogens of *L. lactis* 3107 harboring LC3, TP901-1 or Dub35A. Each of these lysogens exhibits a phage-resistance phenotype mediated through repressor-mediated superinfection immunity (LC3, TP901-1, Dub35A) [32], superinfection exclusion of heterologous phages (LC3, TP901-1) [13,14], or other phage-resistance mechanisms such as potential abortive infection systems (Dub35A). The improved phage-resistance of the generated lysogens highlights the potential benefit of prophage carriage to lactococcal strains and consolidating the notion that prophage carriage among lactococci provides advantages to the host strain. Although *L. lactis* 3107 is not typically a genetically amenable strain and tools for the genetic manipulation of this strain are limited, lysogens were generated readily, suggesting a high degree of genomic plasticity and adaptability in this model strain.

Whereas the repressor-mediated immunity effects were as expected, with LC3lys providing immunity against the P335 phages with homologous repressors, the phage sensitivity profiles of TPlys and Dublys highlighted the likely presence of resistance systems with activity against heterologous phages. This instigated a detailed analysis of the genomes of TP901-1 and Dub35A beyond the lysogeny module for the presence of potential phage-resistance functions. These efforts resulted in the identification of a potential Gp157-like protein that has been implicated in phage-resistance development in Sfi-like phages of *Streptococcus thermophilus* [30]. However, despite multiple attempts to define the source of the heterologous phage resistance observed in 3107-Dublys, it was not identified in the present study. All genes between the integrase- and repressor-encoding genes, and *orf12_Dub35A_*, were cloned and expressed in *L. lactis* 3107, but no significant phage-resistance was observed. This suggests that additional gene(s) are present on the genome of Dub35A that encode such functions or that a combination of such genes may be required for functionality. It also suggests that there remain some novel and, as yet, unidentifiable, phage-resistance genes, and these may be a powerful tool in developing strategies to limit phage proliferation in the dairy fermentation industry.

A notable outcome of the present study is the broad ranging effect of Sie_LC3._ This protein is an identical homologue of the well-characterized protein, Sie_2009_, encoded by the lactococcal temperate phage Tuc2009 [14]. It was previously believed that this (and other similar Sie) system(s) was limited in providing resistance to a subset of skunaviruses [13,14]. However, it is now clear that Sie_LC3_ and its homologous counterparts provide a much broader range of phage-resistance than previously known. It has recently been established that many lactococcal phages recognize and bind to saccharidic receptors [15,17,32,33]. Furthermore, it is suggested that LC3 and TP901-1 pursue distinct DNA injection routes [34]. Lactococcal Sie systems are hypothesized to impede phage DNA injection either by physical interaction with the phage distal tail components(s) or through masking of the DNA injection trigger molecule. The results of the present study suggest that members of the P087 and 949 groups, and those of certain skunaviruses, may follow similar DNA injection pathways to those of LC3, while that of TP901-1 appears to be distinct. Therefore, the LC3 DNA injection route may represent a major pathway followed by a broad array of lactococcal phages.

The foundation study of IL1403 and its prophage-cured derivatives highlighted that prophage-curing resulted, in many cases, in decreased susceptibility to antimicrobial compounds including lysozyme and nisin [9]. Following a reverse approach in which lysogens of three different phages were developed, it was established that, indeed, the presence of prophages increased the susceptibility to lysozyme and nisin; however, the response was lysogen-specific, highlighting the need for targeted studies of a range of model strains to define the disrupting impact of distinct lysogenic phages. Although this sensitivity to anti-microbials may have a detrimental effect on the host, this may also be perceived positively in cases where culture lysis may be associated with the development of flavor or aroma compounds.

During this study, it was attempted to derive “stacked” lysogens in which more than one phage was to be incorporated in the genome of *L. lactis* 3107. Despite numerous attempts, such multi-lysogens could not be generated under the tested conditions and in some cases (3107-TPlys, in particular) spontaneous induction was observed when phage-pressure was applied to the lysogens. This suggests that a given lactococcal strain has the potential to acquire multiple lysogenic phages but also controls the level of carriage to impart the optimal balance of host fitness without compromising its integral growth and phenotypic characteristics. *L. lactis* ssp. *cremoris* strains are widely used in the dairy fermentation industry and because members of the P335 group are among the most frequently encountered in this environment, this study has vast implications for the dairy sector. For example, prophages with greater than 90% sequence identity to TP901-1 (14 strains) and LC3 (18 strains) are present on the genomes of sequenced lactococcal strains with a query coverage of at least 25%, highlighting the direct relevance of this study to a broad range of lactococcal strains harboring similar phages within their genomes. Although considerable analysis remains to be undertaken to understand the complexity of the relationships between lactococci and their temperate phages, this study has highlighted the potential phage-resistance advantages that may be conferred on the host, and that there remain potentially novel phage-resistance systems on the (pro)phage genomes.

## 5. Conclusions

Prophage carriage has long been a contentious issue in the dairy industry. This study provides insights into the ability of lactococcal strains to integrate (pro)phages, enhancing their robustness against phages while limiting the incorporation of additional lysogens where it represents a threat to host fitness and performance. Sie systems present the potential to unlock the current knowledge gap in the mechanisms by which lactococcal phages inject their DNA into the host cell. This study has highlighted that a prevalent mechanism of phage DNA injection among lactococcal may be exemplified by that of LC3. Novel insights into the intricate relationship between *L. lactis* 3107 and its (virulent and temperate) infecting phages have been provided through this study. However, this study also highlights the need for genetic tool development for model and/or industrial strains to facilitate targeted mutagenesis, and for directed prophage-curing efforts, among other approaches, to fully unravel these important and industrially significant interactions.

## Figures and Tables

**Table 1:** Characteristics of predicted prophage-encoding regions of *L. lactis* 3107.

Prophage Region	Status *	Region Length (kb)	Coordinates on 3107 Genome	AT% Content	Related (pro)Phages (%id/%Coverage)
1	Intact	42.6	848,137–890,802	64.09	TP901-1 (93/47)
2	Questionable	16	1,610,756–1,626,788	64.90	TP712 (99.95/51)
3	Intact	48.8	1,936,000–1,984,883	65.45	bIL309 (96.94/23)
4	Incomplete	28.9	2,055,457–2,084,400	64.93	bIL310 (86.95/18)
5	Incomplete ^a^	13.1	2,128,356–2,141,525	65.93	

* Status based on Phaster output. ^a^ Based on manual inspection, prophage region 5 is disregarded as a prophage component.

## Supplementary Materials

The following are available online at https://www.mdpi.com/2076-2607/8/11/1685/s1, Table S1. Bacteria, bacteriophages and plasmids used in this study, Table S2. Efficiency of plaquing of *L. lactis* 3107-infecting phages on lysogens of LC3, TP901-1 and Dub35A, respectively, Table S3. Efficiency of plaquing of phages on *L. lactis* 3107 derivatives expressing lysogeny-related genes of LC3 and TP901-1.
