# Supplementary material for: Lysogenization of a Lactococcal Host with Three Distinct Temperate Phages Provides Homologous and Heterologous Phage Resistance
Source: Microorganisms. 2020 Oct 29;8(11):1685. doi: 10.3390/microorganisms8111685 (PMC7693887; doi:10.3390/microorganisms8111685)
Supplement: Supplementary file 1 [file microorganisms-08-01685-s001.pdf]

Table S1. Bacteria, bacteriophages and plasmids used in this study.

**Bacterial Strains**	**Features**	**Source**
L. lactis subsp. *cremoris*		
3107	Host to TP901-1, LC3, Dub35A, C41431, 63301, 86501, 50101, 62601, 66901, P087, 949 and WRP3	[1]
3107-LC3lys	*L. lactis* 3107 lysogen with LC3 integrated	This study
3107-TPlys	*L. lactis* 3107 lysogen with TP901-1 integrated	This study
3107-Dub35lys	*L. lactis* 3107 lysogen with Dub35A integrated	This study
E. coli EC101	Cloning host	[2]
**Bacteriophages**		
TP901-1	Temperate P335 group phage induced from *L. lactis* subsp. *cremoris* 901-1	[3]
LC3	Temperate P335 group phage grown lytically on *L. lactis* 3107	[4]
Dub35A	P335 phage	[5]
63301	P335 phage	[5]
86501	P335 phage	[5]
50101	P335 phage	[5]
C41431	P335 phage	[5]
62601	Skunavirus (936 phage)	[6]
66901	Skunavirus (936 phage)	[6]
949	949 phage	[7]
WRP3	949 phage	[8]
P087	P087 phage	[9]
**Plasmids**		
pNZ44	*E. coli*–*L. lactis* high copy number shuttle vector containing the constitutive P44 promoter; Cm^r	[10]
pNZ*rep*$_{LC3}$	pNZ44 vector harbouring repressor gene of LC3	This study
pNZ*sie*$_{LC3}$	pNZ44 vector harbouring the *sie* gene of LC3	This study
pNZ*orf3*$_{Dub35A}$	pNZ44 vector harbouring *orf3* of Dub35A	This study
pNZ*orf4*$_{Dub35A}$	pNZ44 vector harbouring *orf4* of Dub35A	This study
pNZ*orf5*$_{Dub35A}$	pNZ44 vector harbouring *orf5* of Dub35A	This study
pNZ*orf4/5*$_{Dub35A}$	pNZ44 vector harbouring *orfs 4 & 5* of Dub35A	This study
pNZ*orf12*$_{Dub35A}$	pNZ44 vector harbouring *orf12*of Dub35A	This study
pNZ*orf2/3*$_{TP901-1}$	pNZ44 vector harbouring *orfs 2 & 3* of TP901-1	This study

Table S2. Efficiency of plaquing of *L. lactis* 3107-infecting phages on lysogens of LC3, TP901-1 and Dub35A, respectively.

Phage	3107	3107-LC3lys	3107-TPLys	3107-DubLys
TP901-1 (T)	1	$2.20 \times 10^{-1} \pm 1.38 \times 10^{-2}$	$6.34 \times 10^{-7} \pm 1.55 \times 10^{-8}$	$6.79 \times 10^{-8} \pm 5.91 \times 10^{-8}$
LC3 (T)	1	$\leq 2.97 \times 10^{-9}$	$7.41 \times 10^{-1} \pm 5.1 \times 10^{-1}$	$8.54 \times 10^{-2} \pm 3.0 \times 10^{-1}$
Dub35A (T)	1	$1.17 \times 10^{-1} \pm 2.50 \times 10^{-2}$	$2.70 \times 10^{-1} \pm 1.01 \times 10^{-1}$	$\leq 4.35 \times 10^{-9}$
C41431 (V)	1	$2.27 \times 10^{-1} \pm 2.21 \times 10^{-2}$	$1.67 \pm 6.67 \times 10^{-1}$	$\leq 1.35 \times 10^{-9}$
50101 (T)	1	$1.17 \times 10^{-7} \pm 3.82 \times 10^{-8}$	$6.20 \times 10^{-1} \pm 2.1 \times 10^{-1}$	2.5 ± 4.96
63301 (T)	1	1.04 ± 0.16	$9.16 \times 10^{-1} \pm 9.05 \times 10^{-1}$	$9.09 \times 10^{-1} \pm 8.31 \times 10^{-1}$
86501 (T)	1	$1.67 \times 10^{-8} \pm 1.05 \times 10^{-9}$	$6.00 \times 10^{-2} \pm 5.10 \times 10^{-1}$	$2.56 \times 10^{-1} \pm 4.51 \times 10^{-1}$
62601	1	$2.95 \times 10^{-1} \pm 6.76 \times 10^{-2}$	$5.70 \times 10^{-1} \pm 1.07 \times 10^{-1}$	$1.73 \times 10^{-7} \pm 1.33 \times 10^{-8}$
66901	1	$2.46 \times 10^{-1} \pm 1.14 \times 10^{-2}$	$1.76 \times 10^{-5} \pm 2.54 \times 10^{-6}$	$\leq 7.90 \times 10^{-9}$
949	1	$7.05 \times 10^{-1} \pm 3.62 \times 10^{-2}$	$1.54 \times 10^{-1} \pm 1.29 \times 10^{-1}$	$9.32 \times 10^{-2} \pm 3.86 \times 10^{-3}$
WRP3	1	$6.41 \times 10^{-1} \pm 2.03 \times 10^{-2}$	$1.53 \times 10^{-6} \pm 1.08 \times 10^{-6}$	$8.00 \times 10^{-7} \pm 5.88 \times 10^{-7}$
P087	1	1.13 ± 0.18	$\leq 1.60 \times 10^{-9}$	$\leq 1.60 \times 10^{-9}$

Table S3. Efficiency of plaquing of phages on *L. lactis* 3107 derivatives expressing lysogeny-related genes of LC3 and TP901-1.

Phage species	*L. lactis* subsp. *cremoris*	3107	3107::pNZ44	3107::pNZ*sie*$_{LC3}$	3107::pNZ*rep*$_{LC3}$	3107::pNZ*orf2/3*$_{TP901-1}$
P335	LC3	1	0.87 ± 0.05	$\leq 10^{-8}$	$\leq 10^{-8}$	0.92 ± 0.10
	50101	1	0.87 ± 0.03	$\leq 10^{-8}$	$\leq 10^{-8}$	0.58 ± 0.07
	63301	1	0.83 ± 0.02	$\leq 10^{-8}$	1.87 ± 0.14	0.83 ± 0.05
	86501	1	0.87 ± 0.11	$\leq 10^{-8}$	$\leq 10^{-8}$	0.02 ± 0.01
	Dub35A	1	0.95 ± 0.09	$\leq 10^{-8}$	0.99 ± 0.08	$0.01 \pm 2.0 \times 10^{-3}$
	TP901-1	1	0.80 ± 0.04	0.02 ± 0.01	1.02 ± 0.09	0.98 ± 0.06
	C41431	1	0.84 ± 0.10	$\leq 10^{-8}$	1.07 ± 0.08	0.66 ± 0.11
Skunaviruses (936)	62601	1	0.81 ± 0.12	$\leq 10^{-9}$	1.52 ± 0.25	$6.70 \times 10^{-4} \pm 3.20 \times 10^{-5}$
	66901	1	0.70 ± 0.06	$\leq 10^{-9}$	1.20 ± 0.17	$2.15 \times 10^{-6} \pm 3.1 \times 10^{-7}$
949	949	1	0.86 ± 0.06	$\leq 10^{-8}$	1.68 ± 0.22	0.12 ± 0.02
	WRP3	1	0.81 ± 0.09	$\leq 10^{-8}$	1.92 ± 0.13	$\leq 9.00 \times 10^{-11}$
P087	P087	1	0.92 ± 0.08	$\leq 10^{-9}$	1.14 ± 0.12	$\leq 1.60 \times 10^{-9}$

1. Braun Jr, Volkmar, Stefan Hertwig, Horst Neve, Arnold Geis, and Michael Teuber. "Taxonomic Differentiation of Bacteriophages of *Lactococcus Lactis* by Electron Microscopy, DNA-DNA Hybridization, and Protein Profiles." *Microbiology* 135, no. 9 (1989): 2551-60.
2. Sambrook, Joseph, and David W Russell. "Transformation of *E. Coli* by Electroporation." *Cold Spring Harbor Protocols* 2006, no. 1 (2006): pdb. prot3933.
3. Christiansen, Bettina, MG Johnsen, E Stenby, FK Vogensen, and K Hammer. "Characterization of the Lactococcal Temperate Phage Tp901-1 and Its Site-Specific Integration." *Journal of bacteriology* 176, no. 4 (1994): 1069-76.
4. Lillehaug, D, B Lindqvist, and NK Birkeland. "Characterization of Philc3, a *Lactococcus Lactis* Subsp. *Cremoris* Temperature Bacteriophage with Cohesive Single-Stranded DNA Ends." *Applied and environmental microbiology* 57, no. 11 (1991): 3206-11.
5. Oliveira, Joana, Jennifer Mahony, Gabriele Andrea Lugli, Laurens Hanemaaijer, Thijs Kouwen, Marco Ventura, and Douwe van Sinderen. "Genome Sequences of Eight Prophages Isolated from *Lactococcus Lactis* Dairy Strains." *Genome announcements* 4, no. 6 (2016): e00906-16.
6. Oliveira, J., J. Mahony, L. Hanemaaijer, Trhm Kouwen, and D. van Sinderen. "Biodiversity of Bacteriophages Infecting *Lactococcus Lactis* Starter Cultures." *J Dairy Sci* 101, no. 1 (2018): 96-105.
7. Samson, Julie E, and Sylvain Moineau. "Characterization of *Lactococcus Lactis* Phage 949 and Comparison with Other Lactococcal Phages." *Applied and environmental microbiology* 76, no. 20 (2010): 6843-52.
8. Mahony, J., W. Randazzo, H. Neve, L. Settanni, and D. Sinderen. "Lactococcal 949 Group Phages Recognize a Carbohydrate Receptor on the Host Cell Surface." *Appl Environ Microbiol* 81 (2015).
9. Villion, M., M. C. Chopin, H. Deveau, S. D. Ehrlich, S. Moineau, and A. Chopin. "P087, a Lactococcal Phage with a Morphogenesis Module Similar to an *Enterococcus Faecalis* Prophage." *Virology* 388, no. 1 (2009): 49-56.
10. McGrath, Stephen, Gerald F Fitzgerald, and Douwe Van Sinderen. "Improvement and Optimization of Two Engineered Phage Resistance Mechanisms in *Lactococcus Lactis*." *Applied and environmental microbiology* 67, no. 2 (2001): 608-16.
